# Autoregulation of bacterial gene expression: lessons from the MazEF toxin–antitoxin system

**DOI:** 10.1007/s00294-018-0879-8

**Published:** 2018-08-21

**Authors:** Nela Nikolic

**Affiliations:** Institute of Science and Technology (IST) Austria, 3400 Klosterneuburg, Austria

**Keywords:** Toxin–antitoxin system, MazF, Autoregulation, Gene expression, Feedback, Phenotypic heterogeneity

## Abstract

Autoregulation is the direct modulation of gene expression by the product of the corresponding gene. Autoregulation of bacterial gene expression has been mostly studied at the transcriptional level, when a protein acts as the cognate transcriptional repressor. A recent study investigating dynamics of the bacterial toxin–antitoxin MazEF system has shown how autoregulation at both the transcriptional and post-transcriptional levels affects the heterogeneity of *Escherichia coli* populations. Toxin–antitoxin systems hold a crucial but still elusive part in bacterial response to stress. This perspective highlights how these modules can also serve as a great model system for investigating basic concepts in gene regulation. However, as the genomic background and environmental conditions substantially influence toxin activation, it is important to study (auto)regulation of toxin–antitoxin systems in well-defined setups as well as in conditions that resemble the environmental niche.

## Overview of toxin–antitoxin systems

Toxin–antitoxin (TA) modules are highly versatile systems, generally widespread in prokaryotic genomes (Gerdes et al. 2005; Van Melderen 2010; Yamaguchi and Inouye 2011; Gerdes 2012; Rocker and Meinhart 2016; Hõrak and Tamman 2017). Toxin activation during adverse conditions inhibits fundamental cellular processes, such as replication, translation, and cell wall synthesis, thereby reducing bacterial growth. TA systems are categorized into at least six different types, depending on how the antitoxin neutralizes expression and/or activity of the toxin (Harms et al. 2018). The model *Escherichia coli* K-12 MG1655 strain has at least 37 TA loci in total, with at least 10 toxins characterized as type II toxins with RNA-degrading activity, i.e., endoribonucleases. Type II endoribonucleases can be active independent of ribosomes or in a ribosome-dependent manner, and few of them cleave RNA sequence specifically (Masuda and Inouye 2017; Harms et al. 2018). Stressful conditions promote the activity of the Clp and Lon proteases that degrade type II antitoxins (Goeders and Van Melderen 2014).

In general, the MazEF system is one of the most studied type II TA modules. The MazE antitoxin directly binds to the MazF toxin and forms a stable complex thereby neutralizing the MazF activity (Kamada et al. 2003). It has been biochemically shown that the MazE antitoxin is degraded by ClpAP (Aizenman et al. 1996), while the Lon protease has additionally been implicated in the *mazEF* regulation (Christensen et al. 2003; Tripathi et al. 2014). Upon MazE proteolysis, the MazF toxin is liberated from the complex, and degrades single-stranded RNA at specific sequences. MazF of *E. coli* recognizes an ACA sequence as the core cleavage site (Zhang et al. 2003); however, specific nucleotides flanking the core sequence, as well as the RNA secondary structure contribute to the efficiency of MazF-mediated RNA cleavage (Culviner and Laub 2018).

## Transcriptional autoregulation of TA systems

Transcriptional autoregulation has been biochemically described for many type II TA systems, usually through the mechanism of conditional cooperativity (Overgaard et al. 2008; Garcia-Pino et al. 2010). This mechanism defines the state of transcription given the molar ratio of toxin to antitoxin within a cell (Page and Peti 2016). When the ratio is similar, the toxin acts as a co-repressor within the toxin–antitoxin complex, and the complex strongly represses transcription. When the toxin to antitoxin ratio is in favor of the antitoxin, the antitoxin alone acts a weaker transcriptional repressor. In conditions that promote degradation of the antitoxin, toxin to antitoxin ratio is in favor of the toxin, which leads to the de-repression of transcription of the TA module (Gelens et al. 2013; Cataudella et al. 2013; Vandervelde et al. 2016).

Conditional cooperativity has also been suggested for the autoregulation of *mazEF* expression (Zorzini et al. 2015). During exponential growth, the expression of the *mazEF* module is strongly repressed (Marianovsky et al. 2001; Nikolic et al. 2017). Analysis of the *mazEF* promoter activity in a *mazF* deletion strain, when the toxin to antitoxin ratio is artificially shifted in favor of the MazE antitoxin, shows weak repression of the *mazEF* operon. Adverse conditions promote modest transcriptional activation and potential de-repression of the *mazEF* module (Muthuramalingam et al. 2016; Shan et al. 2017; Nikolic et al. 2017), suggesting transient de novo synthesis of the toxin and antitoxin proteins at low levels. As de-repression has been directly shown only for high toxin to antitoxin ratios that are seemingly not measured in vivo, a mechanistic explanation of possible conditional cooperativity in the *mazEF* regulation is still elusive (Zorzini et al. 2015).

## Post-transcriptional autoregulation of TA systems

RNA-based regulation of TA systems has been mostly described for type I toxins (Page and Peti 2016; Berghoff et al. 2017; Berghoff and Wagner 2017). However, type II TA systems can also be regulated at the RNA level, as recently shown in the case of post-transcriptional autoregulation of *mazEF* expression (Nikolic et al. 2018). During ectopic *mazF* expression, the MazF toxin cleaves the *mazF* mRNA at ACA sites, which has direct consequences on the behavior of single cells. Single-cell analysis indicated that the cell length fluctuates substantially during conditions that promote MazF activation. Cells elongate and become filamentous, and divide irregularly during *mazF* overexpression, which suggests that mRNAs of proteins involved in cell elongation and division are targeted by MazF (Schifano et al. 2014; Sauert et al. 2016; Venturelli et al. 2017). A mathematical model showed that MazF-mediated cleavage of the *mazEF* mRNA can result in stochastic pulsed excitations of MazF levels in single cells, suggesting that the observed cell length fluctuations are a consequence of the fluctuations in MazF levels during stress. Further analysis indicated that the frequency of the toxin spikes and the amount of free toxin released during the pulses depend on the amount of stress (Vet, Vandervelde and Gelens, unpublished). Overall, the majority of the known type II TA systems are RNA-degrading enzymes, degrading RNA while bound to the ribosome, or in a ribosome-independent manner, such as the MazF toxin (Masuda and Inouye 2017; Harms et al. 2018). Thus, an open question remains: Do other RNA-degrading toxins also autoregulate their expression by degrading their cognate mRNAs? And more importantly, how does post-transcriptional autoregulation of type II TA systems influence the fate of bacterial cells in stressful conditions, for instance during antibiotic treatment or nutrient starvation?

## Autoregulatory circuits and feedback loops in TA systems

In a one-player negative feedback system, a protein represses its own expression. One prominent example is the regulation of *tetR* expression by the TetR transcriptional repressor (Becskei and Serrano 2000; Rosenfeld et al. 2002). Autoregulation of the *mazEF* expression contains negative and positive feedback. Negative feedback manifests when the MazE–MazF complex represses transcription of the operon (Marianovsky et al. 2001). At the post-transcriptional level, complete *mazEF* mRNA degradation by MazF prevents de novo synthesis of the toxin and antitoxin proteins, and favors long-lived MazF toxin over short-lived MazE antitoxin, thus resulting in a positive feedback loop (Nikolic et al. 2018). Another recent model has analyzed TA regulatory circuits in a setting that can be generally applied to any stressful condition that results in growth reduction and antitoxin degradation (Tian et al. 2017). The modeled TA locus, which consists of a toxin gene downstream of an antitoxin gene, is transcribed into a bicistronic mRNA. Toxin-mediated cleavage of the toxin part of the mRNA leads to the antitoxin protein synthesis, thus manifesting as a negative feedback. As the toxin module in this model contains no ribosome binding site, cleavage in the antitoxin part of the mRNA prevents synthesis of both antitoxin and toxin proteins, thus manifesting as a positive feedback (Tian et al. 2017). In general, the MazEF system contains at least two players and several interactions, thus feedback loops possibly operating at different levels and different time scales. Distinct regulatory circuits within this TA system can be monitored in a synthetic experimental setup by adjusting levels of toxin and antitoxin production.

## Genomic background and environmental cues as determinants of TA regulation

Even though there is substantial information about TA systems in non-pathogenic, laboratory strains of *E. coli*, less is known about TA systems in pathogenic *E. coli*. One reason for this lack of knowledge is that TA loci are acquired through horizontal gene transfer, and thus are not conserved among different isolates belonging to the same bacterial species (Fiedoruk et al. 2015; Ramisetty and Santhosh 2015); for instance, the *mazEF* locus is present in the non-pathogenic K-12 MG1655 *E. coli* strain, but not in the uropathogenic CFT073 strain (Norton and Mulvey 2012). Moreover, the underlying genomic background can shape the response of individual TA systems during stress. Because TA systems form complex regulatory networks by cross-activating each other (Yamaguchi and Inouye 2011; Kasari et al. 2013; Wessner et al. 2015), it is important to study the activity of individual toxins given the strain-specific genomic background. Sequence-identical toxin homologs from different strains could thus differ in their efficacy and the time needed for their activation.

Besides contribution of genomic background to regulation and autoregulation of TA operons, another factor is crucial when investigating TA systems—environmental signals (Ramisetty and Santhosh 2017; Hõrak and Tamman 2017). Based on analysis of a commonly used laboratory strain, deletion of a single TA locus does not influence competiveness of *E. coli* populations in a culture flask (Tsilibaris et al. 2007). However, a previous study has shown that individual TA systems of an uropathogenic *E. coli* strain indeed provide growth advantage during niche-specific colonization (Norton and Mulvey 2012). Furthermore, TA modules of *Salmonella* promote formation of intracellular persisters upon phagocytosis by macrophages (Helaine et al. 2014). These studies highlight the strong need to investigate the regulation and roles of TA systems in pathogenic bacteria during infection, as bacteria do not experience cues from their environmental niches while growing in the laboratory medium. Little is known about signals that trigger TA systems in general; however, it is assumed that TA systems respond to stressful conditions (Aizenman et al. 1996; Ramisetty and Santhosh 2017; Harms et al. 2018; Goormaghtigh et al. 2018). An essential question still remains: What are actual environmental signals that promote activation of TA systems in the bacterial natural niches?

## Experimental setups for analysis of TA systems

As genomic background and environmental cues are determinants of TA regulation, it is important to pay attention to the strain genotype and to note exact conditions of laboratory cultivation when investigating TA (auto)regulation in commonly used *E. coli* laboratory strains. When planning the experimental setup, one needs to consider several aspects, for instance, to carefully decide on which *E. coli* laboratory strain to use: the MG1655 and MC4100 strains differ from each other significantly in their genotypes (Peters et al. 2003; Kolodkin-Gal and Engelberg-Kulka 2008) (e.g., compare Vesper et al. 2011 with Culviner and Laub 2018). Experiments can have different outcomes depending on whether the chosen strain has a functional *relA* locus (Tsilibaris et al. 2007), or if the strain carries prophage insertions or other genomic rearrangements that potentially influence the TA response (Harms et al. 2017; Goormaghtigh et al. 2018). Furthermore, different ectopic expression systems can produce different toxin levels based on the strength of the inducible promoter, as well as whether the expression system is inserted into the chromosome, or based on a medium-copy or a high-copy plasmid. For instance, mild ectopic *mazF* expression results in slight growth reduction (Nikolic et al. 2017), while ectopic *mazF* overexpression from a medium-copy plasmid results in stronger growth reduction and promotes formation of phenotypically distinct subpopulations (Nikolic et al. 2018). Moreover, *mazF* overexpression differently affects cellular physiology based on the presence or absence of the MazE antitoxin that buffers MazF toxic effects (Nikolic et al. 2018). It is likewise necessary to report exact media composition used in different experiments, as well as whether bacterial populations are cultivated with or without shaking. Published experimental protocols also indicate adding the inducer of *mazF* expression in bacterial cultures of different optical densities, e.g., during early exponential phase (Tsilibaris et al. 2007; Nikolic et al. 2018), or mid-exponential phase (Amitai et al. 2004; Kolodkin-Gal et al. 2009). However, it is probable that the bacterial growth phase affects toxin activation due to, for instance, interaction of TA systems with mechanisms that are active during shifts in nutrient composition or during other environmental changes (Battesti et al. 2011; Wang and Wood 2011).

Measuring toxin and antitoxin levels in single cells in vivo is a challenging task. As toxin and antitoxin proteins form complexes, tagging them with fluorescent reporters would most certainly decrease stability of the complexes and prevent efficient toxin neutralization by the antitoxin. In addition, tagging toxins with fluorescent proteins could interfere with the toxin activity (Berghoff et al. 2017). Another approach is using transcriptional fluorescent reporter systems by fusing the promoter of a TA operon to a fluorescent gene reporter. However, several studies have reported that such analysis suggests weak to moderate de novo transcription of TA modules in conditions that presumably promote their activation (Shan et al. 2017; Nikolic et al. 2017; Goormaghtigh et al. 2018). One reason for the modest fluorescent readout is that many stress conditions interfere with translation, thus lowering protein synthesis, and subsequently decreasing the production of fluorescent proteins. Even though de novo transcription of TA modules is indicative of an imbalance of the toxin to antitoxin ratio and transient occurrence of free toxin proteins, transcriptional fluorescent reporters for TA systems do not directly report on toxin activation. It is therefore essential to build reporter systems that are independent of translation, and that report on toxin activation in real time.

## Toxin activation influences phenotypic heterogeneity

Phenotypic heterogeneity in clonal bacterial populations can be measured between cells at a designated time point (cell-to-cell variation), or as variability in phenotypic traits of the individual cells in time (temporal variation) (Ackermann 2015). In general, negative feedback at the transcriptional level reduces cell-to-cell variation (Becskei and Serrano 2000); however, it has been suggested that negative feedback at the post-transcriptional level reduces cell-to-cell variation more efficiently than a transcriptional regulation feedback (Singh 2011). Additional interactions can change the nature of the feedback system (Ananthasubramaniam and Herzel 2014); for instance, positive feedback interactions may arise within a negative feedback motif (Bokes et al. 2018; Nikolic et al. 2018). Positive feedback amplifies phenotypic heterogeneity, and can generate subpopulations of cells with different phenotypic states. For further discussion on how different feedback loops influence cellular processes in bacterial cells, see (Smits et al. 2006; Maheshri and O’Shea 2007; Ackermann 2015).

Several previous studies indicate that the majority of chromosomally encoded type II TA systems elicit phenotypic heterogeneity in stressed bacterial populations by promoting variation in gene expression, cell size, and growth rate (Klumpp et al. 2009; Kasari et al. 2010; Nikolic et al. 2017, 2018). During ectopic MazF activation, the extent of cell-to-cell variation was slightly reduced when MazF was able to degrade its cognate transcript. Interestingly, MazF-mediated cleavage of the *mazEF* transcript induced pulse-like behavior and increased temporal variability in MazF levels during stress (Nikolic et al. 2018). Why would the same post-transcriptional autoregulatory circuit promote lower cell-to-cell phenotypic variation and higher temporal variability? This question requires more detailed experimental and theoretical analysis, as synthetic setups can influence the nature of feedback loops, and favor specific feedback architectures.

## Current questions about *mazEF* (auto)regulation

The *mazEF* autoregulation is a complex mechanism attained by several protein–protein, protein–mRNA and protein–DNA interactions. Autoregulation of *mazEF* expression at the transcriptional and post-transcriptional level is responsible for fluctuations in MazF levels during ectopic stress (Nikolic et al. 2018). However, the *mazG* and *relA* transcripts are parts of the polycistronic mRNA together with *mazE* and *mazF* (Gama-Castro et al. 2016). MazG is an enzyme with pyrophosphohydrolase activity (Zhang and Inouye 2002), and it is considered to cause depletion of ppGpp (Gross et al. 2006). ppGpp is an alarmone that modulates gene expression during amino acid starvation, and it is synthesized by RelA (Dalebroux and Swanson 2012). Elucidating whether MazG plays a role in the autoregulation of the *mazEFG* transcriptional unit (Goormaghtigh et al. 2018), as well as how transcription of the *relA-mazEF* unit (Gama-Castro et al. 2016) affects *mazEF* expression, will provide necessary details to obtain a full picture of autoregulatory processes. Stress response mechanisms might additionally regulate *mazEF* expression (Battesti et al. 2011; Wang and Wood 2011). Furthermore, the MazF toxin can be inactivated by the T4 bacteriophage-dependent ADP-ribosylation (Alawneh et al. 2016; Otsuka 2016), which raises the question if MazF is chemically modified by other post-translational mechanisms. Likewise, it is still elusive whether other TA systems cross-regulate MazF activity and *mazEF* expression, and to what extent (Yamaguchi and Inouye 2011; Kasari et al. 2013; Wessner et al. 2015). Finally, investigating (auto)regulation of *mazEF* expression during physiological induction of the toxin will further determine the importance and impact on this TA system on the behavior of bacterial cells.
